# What Clinicians Should Tell Patients About Wearable Devices and Data Privacy: A Narrative Review

**DOI:** 10.7759/cureus.81167

**Published:** 2025-03-25

**Authors:** Joseph V Pergolizzi, Jo Ann K LeQuang, Salah N El-Tallawy, Giustino Varrassi

**Affiliations:** 1 Pain Medicine, NEMA Research, Inc., Naples, USA; 2 Scientific Communications, NEMA Research, Inc., Naples, USA; 3 Anesthesia and Pain Management, Faculty of Medicine, Minia University, Minia, EGY; 4 Anesthesia and Pain Management, National Cancer Institute, Cairo University, Cairo, EGY; 5 Anesthesia, King Khalid University Hospital, Riyadh, SAU; 6 Medicine, King Saud University, Riyadh, SAU; 7 Pain Medicine, Fondazione Paolo Procacci, Rome, ITA

**Keywords:** cybersecurity, ethical implications, health app, mobile health app, patient data privacy, smart health wearable, wearable devices, wearable technology

## Abstract

The recent growth of wearable medical device technology in fitness trackers, smartwatches, smartphone apps, and patient monitoring systems has created people-generated health data (PGHD) that may benefit medical science with large amounts of continuous real-world data. The prevalence of these devices speaks to their broad popularity and user-friendliness and may lead us one day to a more fully “connected healthcare system.” Meanwhile, data security, confidentiality, and privacy issues have emerged in these hackable systems. Despite the promise of anonymized data, data can sometimes be re-identified. However, even without that step, data breaches may reveal information (name, address, date of birth, social security number, and so on) sufficient for identity theft. Clinicians are often asked about the utility and value of wearable devices or monitors. Still, most are unaware that data from these systems may be transmitted, stored, and even sold without the user’s specific knowledge. Despite the confidentiality of medical information, cybersecurity surrounding wearables and monitors remains relatively lax, making them comparatively easy targets for cyber villains. It is also important that efforts be made to make PGHD more secure since medical data may be of great value to telehealth applications and AI-physician assistants. Clinicians should actively inform patients about the risks and benefits of wearables and similar devices.

## Introduction and background

Wearable medical monitors and devices (“wearables”), smartphone applications, smart watches, fitness trackers, and similar systems can detect, monitor, and record health-related information and sometimes relay specific alerts to clinics or healthcare professionals. Some wearables are available over the counter to guide individuals in health-related activities such as diet or exercise plans [1]. Implantable devices such as pacemakers may offer remote monitoring and alert systems. There are also hand-held electrocardiography devices and glucose monitors. The market for wearables and similar devices has been estimated to be worth $12 billion in 2023 and is expected to increase substantially in the coming decade [1,2]. The prevalence of widely accepted, user-friendly wearables for the healthcare system may facilitate a more fully “connected healthcare” system that links electronic medical records to wearable systems and monitors [3]. With vigorous sales proving their popularity, people who use these devices often report a sense of empowerment [4]. Whether that empowerment is genuine or illusory is another issue [5].

Wearables may be used for health and lifestyle monitoring, tracking fitness activities, ensuring safety, managing chronic conditions such as heart disease or diabetes, supporting diagnostics, aiding mental health, and facilitating rehabilitation efforts. The ability of wearables to disrupt our current healthcare system and drive positive change is evident [6,7]. Wearables may help stretch already strained medical resources by expanding access to regions with limited healthcare services [8-10]. From piezoelectric tattoo-like films that adhere to the skin's surface to electronic socks, from smartwatches to injectable cardiac monitors, the range of products is imaginative and exciting, particularly for people with a proactive approach to self-care and an openness to technological innovation [11].

Wearables shift the paradigm in healthcare away from the intimacy of occasional face-to-face consultation with a clinical expert to a more continuous, patient-centric, and participatory model. Wearables, fitness trackers, smartphone apps, monitors, and similar systems gather a wealth of person-generated health data (PGHD). Yet, few people who use these devices know that PGHD is inherently valuable beyond its informational content to the user. With the plethora of wearable innovations currently available and coming to market, one important consideration is a legal and ethical framework regarding how this PGHD and other data are stored, shared, aggregated, and utilized, and by whom and for what purpose [12].

While individuals may start using wearables without the benefit of physician advice, others consult healthcare professionals to help guide device selection. Patients sometimes bring wearable-generated reports with them to the clinic. Just as the clinical team works with patients to improve health literacy, discussing data privacy, confidentiality, and security associated with wearables is important. The wearable companies publish this information in their "terms and conditions" but make no particular effort to ensure patients understand it. The result is that people using wearables may be unaware of how their data may be used or the risks associated with data breaches. Many individuals incorporate technology into their everyday routines long before considering the consequences of privacy, data sharing, data breaches, security, identity protection, and confidentiality. However, at most, clinicians can inform patients about data privacy but cannot guarantee it.

This narrative review aims to aid clinicians who may need to discuss the issues of data security and patient privacy with patients who have used or are contemplating using wearables. Google Scholar and PubMed databases were searched for keywords related to wearable data privacy. Bibliographies of relevant articles were also searched. The vast number of devices and applications made reporting on specific devices or even device categories impossible in a short review. This review aims to describe how patients understand privacy issues with wearable trackers, fitness monitors, and similar products and ways clinicians might raise health literacy on these important topics. In this article, the term “wearable” will include not only trackers, smartwatches, and sensor-driven systems but also applications, monitors, and other systems that produce PGHD, including monitoring systems embedded in implantable systems such as cardiac devices and infusion pumps. The field is experiencing rapid development and a proliferation of new devices, and collective terms are not yet readily defined for these many systems.

## Review

The evolution of how medical data are captured, organized, curated, reported, displayed, and utilized has been rapid and enormous. For centuries, clinicians collected health information in private, face-to-face sessions with patients, recording the information on paper or capturing images on film. Electronic health records brought with them digitization, improving the portability of records and allowing greater versatility in capturing and sending images. However, data were mostly obtained during individual, in-clinic sessions between a patient and a healthcare professional face-to-face. By collecting PGHD rather than data self-reported by an individual patient or captured in the clinical setting, wearables brought a new source of real-world health-related information to patients, clinics, and the healthcare system.

With wearables, data collection is driven by patients rather than clinicians, and these data can be continuous, extensive, and of good quality [13]. For example, a patient may self-report daily exercise, but a fitness tracker counts the steps or measures the activity. Wearables can collect a range of patient-related data that may be more far-reaching than patients realize. For instance, even simple fitness trackers for healthy individuals may collect other vital information, such as heart rate and respiration, as well as workout schedules, patterns in exercise, geographical locations, and lifestyle habits, such as sleep schedules [14].

How devices collect and transmit data

Wearables either collect data continuously through sensors or other means, or they can collect data on demand (such as recording a workout) or manually through user input. Data are collected and transmitted wirelessly to a user interface so that these data are coherently organized and displayed, such as a sleep app that displays histograms of weekly sleep patterns. Data may also be subsequently transmitted wirelessly to a database or other repository. Wearable owners may have access to all or some archived information for review or in the form of reports. Data may be archived and reported historically as well.

Data transmissions generally rely on wireless or Bluetooth connections, and data are sometimes altered or modified for more efficient transmission. Since data can be vulnerable to breaches during transmission, encryption is often used to safeguard data in transit [15]. While PGHD has considerable value in medical science, hacking or breaching healthcare data is mainly committed to stealing user identities [16]. Wearable data are attractive to cybercriminals because security provisions for such data are not always robust [17,18]. While smartphones and other data transmission systems are widely used to convey financial and private information, the security safeguards and the deviousness of bad actors have increased almost in tandem. Regrettably, medical and patient monitoring systems have not always kept pace [19]. Thus, hacking medical devices can be a way to steal an identity, and one few people consider. A summary of common hacking terms and associated risks appears in Table 1.

**Table 1:** Key terms related to data privacy and cybersecurity listed in alphabetical order Data security terms, hacker terms, and related dangers [15,16,19]

Term	Definition
Authentication	A method of validation of data destination that confirms the proper identity of the data recipient
Breach	A broad term for any act or event where an unauthorized party can access personal, private, or confidential information
Eavesdropping	Real-time interception of private communications
Hard trust	Mechanisms such as authenticity controls, encryption, algorithms, and audits are in place to harden data security
Interruption	Failure of data in transit to reach its intended destination, which may be due to technical problems or a hacker intervention
Malware	The use of dangerous viruses, worms, or other tools to corrupt data once it is in a repository (such as stored on a computer or app)
Message alterations	Changing the content of the data while they are in transit may be the content of the message or the timestamp. Also called data modification
Sniffing	Monitoring every data packet that passes through a certain checkpoint (network)
Soft trust	A personal and often emotion-drive perception of security and safety, often shaped by the brand and social influence of the device
Tapping	Using a hardware device to access data in transit
Traffic analysis attacks	Monitoring data transmissions from a wearable and a smartphone or software app to identify users or detect their activities
Virtual private network	A private, hidden, and restricted passageway (like a tunnel) through which data can flow

Patients may approach certain wearables with soft trust because of brand familiarity and/or tacit or overt endorsement of the wearable by a clinician or other medical authority. People may be unaware that wearables can be a “point of entry” for identity thieves. For this reason, clinicians must be able to discuss data security with patients, unaware that these devices can be hacked.

For most people using wearables, data storage is the proverbial "black box." Wearable owners do not usually know where their data are stored, how they are stored, who stores them, who has access to them, and what is being done with them [19]. Some individuals may be unaware that the data exists outside of their devices. Most wearable owners may not consider their data valuable to outsiders. Knowingly or unknowingly, most people take a leap of faith with these wearables, assuming their personal information and medical data are properly secured following the law and local regulations. While safeguards exist to protect personal data in all spheres of life, breaches are common. In 2023, over 100 million Americans were affected by a data breach, data leakage, or exposed data; it would be difficult to find an American not directly or indirectly affected by a cyberattack [20].

A key concern with data storage is the ability of data to cross state and even country lines, in which case the laws of the destination locality govern how the data are to be handled [21]. Regulations, laws, and practices of data storage and data protection can vary markedly among countries, so data collected under strict provisions of California law may be transmitted for storage to India, where different and often more lax regulations would prevail [22]. Even if they were aware of this translocation, users may be unable to prevent their data from being moved to other locations. Users would have difficulty ascertaining where their data is stored. Compounding the problem, the same data may be placed in multiple repositories.

A typical wearable collects PGHD, transmits these data to an application (for user review and examination), and sends encrypted data for storage to a company-owned database. Hacks and data breaches of that database can expose all data, including personal identities [23]. An important example of the risks of how vulnerable seemingly innocuous devices can be is the case of Strava, a fitness wearable that uses heat maps to track activity patterns. In the Syrian war zone, the Pentagon discovered that a hack of Strava wearables by U.S. troops revealed to the enemy the positions of U.S. military facilities in Syria and Iraq. The Strava hack even revealed troop movements [24]. While war zone examples may not be relevant to the average patient, it is important to know that the data collected by wearables are stored and may be hacked for nefarious purposes.

How data are used

Besides concerns about hacking, people who use wearables may be unaware that data from their medical devices may be used by third parties without their express permission. PGHD possesses real scientific value and can be sold as such. While legitimate investigators would not utilize PGHD this way, this does not mean there is no market for such data. A survey of 842 people who owned wearables asked respondents if they would be amenable to selling their personal data for various price points. A bimodal distribution occurred, suggesting that the sale of such data might be socially divisive because some respondents considered the data of little value, while others wanted high payments [25]. In a 1,300 American wearable users survey, most were willing to share their health-related data with their clinical team (82%) [26]. Most wearable users are unaware that third parties may also be interested in their data [19].

Ideally, when PGHD arrives at their destination database, they are anonymized or de-identified and arrive in data "packets" so that specific information cannot be traced back to its respective owner. This may give wearable users a false sense of security because retrograde processes can “re-identify” data by finding clues to link anonymized data back to their origin, with success rates as high as 86% for certain cases. The re-identification process needs only to take less than five minutes of live data from the device to use the electrocardiogram, heart rate, respiration rate, gait, or other factors to match allegedly de-identified data with their owner [27]. While this would rarely be used to identify random individuals, it is possible that specific data from a specific user might still be traceable.

Wearables are a medical novum, meaning privacy issues have yet to evolve into nuanced options for device owners. Ideally, wearable owners should be able to opt out of data collection, but not all devices allow this, and not all consumers would demand it. Wearable users have few ways to confirm whether their data are being collected, by whom, and where this information is stored. The U.S. government has few ways to hold companies responsible if they collect data on unwilling subjects [27].

PGDH are often shared, sold, and distributed to third parties without the knowledge or consent of the wearable owners [28]. Although not a wearable system, a good case is the proliferation of DNA-testing services, which hold genetic information on tens of millions of people. These DNA-testing companies are not obliged to follow Health Insurance Portability and Accountability Act (HIPAA) regulations because they are considered neither healthcare providers nor insurance companies. Nevertheless, genomic data stored by these companies are sometimes sold to companies for medical research [29]. To protect the medical public, the Data Sharing Hierarchy (DASH) guideline was compiled following a hierarchy of threats to patient privacy [30]. While offering only a framework, the goal of DASH was to bring a degree of risk management to PGHD collection. A crucial finding of DASH was that as technology improved, risks to patient privacy increased [30]. In other words, new technology brings new risks.

Who owns PGDH?

PGHD offers abundant real-world patient data, and its value is hard to overstate. Nevertheless, PGHD remains inadequately defined and understood, particularly regarding data privacy and security [13]. In the future, PGHD may make digitized clinical trials possible and offer scientific researchers the possibilities of “digital twins” for drug development, personalized medicine, and clinical studies. Medical companies may use PGHD to develop or refine existing products and services. Insurance companies could employ PGHD to more accurately devise beneficial wellness programs and assess risk for various health conditions. Public health organizations can use PGHD for population health assessments, societal health interventions, and contact tracing. Regulatory bodies may use PGHD for post-approval surveillance and safety monitoring and even to contribute to developing medical guidelines [13]. The utility of PGHD may go far beyond this.

The ownership of PGHD poses new questions that individual nations or even individual states within the United States may resolve differently [31]. While an individual can own a wearable device, data ownership is another question rarely addressed in scientific literature. The issue is murky, even going back to the old days of color-coded paper medical files filled with handwritten notes and records. These paper records were considered to be professional medical opinions and thus “original works of authorship” that the physician (or healthcare system) rather than the patient owned [32]. Of course, not all states agree, and New Hampshire, for instance, specifically states that patients own their own healthcare data; other American states are quite silent on the subject [32]. This poses the thorny issue of how a physician or healthcare system that owns healthcare data as “original works of authorship” is then obliged to protect a patient's privacy. For many people and even legal authorities, the issue concerns access, data protection, safety, and ownership.

In the United States, once data are de-identified, HIPAA restrictions no longer apply [32]. Large de-identified datasets can be very valuable and sold to researchers. Owners of de-identified datasets can sell this data, but patients get no remuneration [32]. Most patients do not know that their medical records may contribute to large medical databases bought and sold.

Explaining medical privacy to patients can be challenging because the legal and medical arguments are convoluted and sometimes strange, such as the long-standing trope that patients own “the information” in their records. Still, the provider owns “the record itself.” Digitized data from wearables makes this even more challenging for patients to grasp [33]. It seems that the patient has access to information from the wearable device. Still, that information is owned and can be sold by the device manufacturer without the patient's knowledge or consent.

PGHD, or data from wearable devices, introduced a new legal challenge. Generally, wearable manufacturers stipulate that the data collected by their devices belong to the manufacturer and that the manufacturer is granted broad discretion regarding what to do with them [34]. When Google acquired Fitbit in 2019 for over $2 billion, it was speculated that it was made not to buy the relatively straightforward fitness tracker technology but to acquire the health data of millions of regular Fitbit users [35]. While this information is often disclosed to patients in "terms and conditions," patients may misunderstand data ownership or not consider it important.

Looking ahead: when AI met PGHD

AI requires vast amounts of data to build its large language models, and data from wearables may, in part, feed the machine-building healthcare AI. On the one hand, this may open the door to personalized medicine, cheaper digitized clinical studies, improved patient safety with remote monitoring, and other advantages. Virtual physician assistants are possible with AI. Telehealth applications can be radically expanded and made more personalized [36]. At this point, AI is more of a clinical possibility than a clinical reality. Still, the potential for AI expansion into healthcare is undeniable and will no doubt take shape quickly in the coming years.

The stumbling blocks to greater expansion of AI are currently being sorted out, including privacy rights and technological challenges. Medical data are not collected standardized, and data from wearables are generally offered raw rather than in an efficiently curated package [37]. The development of healthcare AI will require more standardized data collection, better privacy controls, and overcoming clinical reticence to use AI in this way [37]. Google's interest in AI systems and PGHD from Fitbit is a good illustration of how AI will evolve [35].

Healthcare AI will disrupt the healthcare system. AI chatbots could be virtual physician assistants that offer more in-depth telehealth interactions with patients on lifestyle modifications, disease diagnoses, medications, and consultations. Genetic information interpreted with AI may benefit research applications because AI can be used in drug development and digital research studies. Finally, physicians and other healthcare providers can benefit from AI in interpreting complex medical images, reviewing and analyzing data relevant to complex cases, and quickly sorting through vast amounts of data [37]. For example, AI is already used widely in electrocardiogram interpretation when Holter monitors capture hours of tracings [38]. This use of AI saves valuable clinical time while improving accuracy.

Since AI or machine learning is a fast-moving and emerging field, cyber experts must keep pace with privacy concerns as techniques advance [39]. Right now, it appears as if AI will expand more rapidly into healthcare than patient privacy protections. AI seems particularly useful for translating large datasets into meaningful results, but it is imperative that the data can be safeguarded along the way [40].

Patients’ perceptions of data privacy

Wearables are a global phenomenon, but national and cultural distinctions can exist regarding how privacy is defined or valued. In a survey conducted in China (n=2,058), 52% of respondents had experience using some sort of wearable. Still, most did not understand what the device did, how it worked, or privacy issues [14]. In a survey of 1,005 European consumers asked about wearing a smartwatch that would monitor them continuously for evidence of a potential cardiac arrest to facilitate timely intervention, 90% were interested in the technology, and 75% said they would be willing to wear such a watch. Still, their main concerns were privacy, data protection, device reliability, and accessibility of information [41]. In a survey of 550 participants in Germany, 34% said they already wore a smartwatch or some sort of fitness tracker, and 61% were open to data sharing. However, concerns about privacy and data security were raised [42].

Even when wearable manufacturers offer detailed privacy policies, patients and clinicians remain largely unaware of how or why PGHDs are used. Furthermore, wearable owners are often oblivious that, once their data are collected, third parties may further disseminate their data [19]. In other words, the wearable company may sell user data to one company, which may, in turn, share it with a university, granting access to that data to another research organization. The user has no control over these data-sharing cascades. Regulations may be beneficial, but consumer-grade devices like fitness trackers or health applications are not regulated to the same standards as medical devices if they are regulated at all [43].

When a healthcare provider, hospital, clinic, or other suggests wearing wearables, the patient may perceive this as an endorsement and an assurance of data protection [44]. Thus, wearing wearables may require discussing medical privacy, confidentiality, and security. Medical privacy is a complex subject that defies easy definitions, and privacy is often blurred between confidentiality and security (Table 2).

**Table 2:** Open questions involving the privacy, confidentiality, and security in wearables and other health-related devices that collect PGHD PGHD: people-generated health data

	Privacy	Confidentiality	Security
Key questions	Who has access to the information? Under what conditions may the information be accessed?	Are there any limitations on what data may be collected and where/how?	What measures are being used to prevent unauthorized access, use, modification, or dissemination of my data? Are data encrypted?
Domains this affects	How and where are data stored and transmitted? Is personal information (name, address, birthdays, identification) collected?	What third parties (if any) can access the data? What laws are involved if data crosses borders?	How are there to protect against computer hacks, data breaches, and unauthorized data disclosures?
Other issues	Can data collection be prevented in some cases? Are there limits to what type of data is collected?	Can a clinician share data without permission if it is de-identified?	Security authorizes who can access data, but who controls the actions of the authorized users? What limitations (if any) may affect authorized users?
Data owner	Who owns the data?	How does the manufacturer protect the user’s privacy?	How does the system secure the data?
Crucial points to consider	Who may sell the data?	Can the wearable owner limit data collection of particularly sensitive information (mental health issues, pregnancy, cancer)?	What techniques are used for cybersecurity?

Many patients are concerned that sensitive or personal health-related information might be used in ways that could cause discrimination against them, such as information about disease diagnoses, mental health conditions, substance use disorders, and other sensitive topics. Prospective employers, universities, insurance customers, social service offices, and others could be influenced adversely by such information. Minorities, those with mental health or substance use disorders, and transgender persons in particular were afraid that unauthorized use of their data could have negative consequences for them [45]. In a survey of 1,000 patients, 92% of respondents said that they had a right to privacy with their health data and that third parties should not purchase such data. In this survey, 80% of patients wanted to have a way to opt out of sharing their data with companies, and 75% said no health data should be shared without a prior opt-in by the patient [45]. These patients are likely unaware that such data sharing is already happening.

Health literacy of patients on wearable privacy

Most wearables provide "terms and conditions" and a lengthy privacy policy, which is typically difficult for laypeople to understand. Users rarely read such documents, and when wearables use small screens like a watch face or a smartphone screen, the small font can make reading these texts particularly cumbersome [19]. In a convenience sample survey of 106 participants who used some sort of wearable medical or fitness application (45% had a smartphone app, 31% an Apple Watch, and 24% a Fitbit), 53% said they did not know how their device transmitted, stored, labeled, or handled their personal information. Moreover, 28% did not realize that health-related information was confidential or private. Data protection policies were familiar to 52% of respondents; however, 57% did not know what to do or whom to contact if they had questions about their data privacy, confidentiality, or security [19].

Patients should be informed that their data are likely owned by the device manufacturer, who de-identifies and collects these data; patients should also know that manufacturers are very likely to share or sell their data. This can surprise patients who may regard their data as possessing little to no commercial value. Privacy policies and "terms and conditions" may report that wearable data are “de-identified” or aggregated and anonymized. However, this may provide a false sense of security because data can be re-identified under certain conditions. Re-identification is not as difficult as it sounds [46]. Anonymized or de-identified data are important forms of data safety but do not confer absolute protection. Patients should also be aware that genetic testing services have control of their genetic information but are even less regulated than wearables since their service is not considered medical.

Clinicians should inform patients that the theft of medical information is a form of cybercrime mainly used in identity theft; this type of theft of medical information is increasing at a higher rate than other forms of cyberstealing [37]. Users may erroneously believe that all a cyberthief can steal is their fitness data, such as steps walked per day or sleep logs; they may not be aware that device hacks can result in stolen identities, often resulting in financial loss [37]. Medical records may contain names, addresses, dates of birth, social security numbers, and possibly credit card or other personal information. To put this in perspective, from 2005 to 2019, there were 249.9 million data breaches [39]. Users of wearable devices should be alerted that these data security issues affect wearables and credit cards or bank information. However, the latter often have more extensive security measures and entire cybersecurity departments.

Many people own and use wearables with little awareness of legibility, the industry term for informing people that their data are being collected and how they are stored [39]. Most application-based wearables rely on two sets of applications: one on or in the device itself and a companion application that resides on the smartphone or computer of the user. A study of 150 wearable applications found that 28 allowed sensitive information to flow across the applications, making the data vulnerable in transit. An example might be a smartwatch (one device) that tracks heart rates and then sends the data to the owner's laptop for display (second device). Further, in a survey related to this study of 63 wearable users, 66.7% did not know there was a possibility for cross-device sharing of sensitive information [47].

Clinical considerations

Clinicians must be mindful that the patient often perceives their recommendation for using a wearable device or monitor as a recommendation for using this technology and, by implication, the assurance that these devices are safe and protect their data. Therefore, clinicians should recommend devices only after they review the privacy protection information or what privacy considerations might be if they recommend a type of device rather than a specific product. Such recommendations may be a good starting point for a short conversation with the patient about data security.

Physicians should also explain that an individual's personal information may come from third parties, like information held by banks, credit card companies, and other businesses. In many cases, the user will not know who this is or have any way to contact that party. A risk in having data stored by third parties is that data breaches can facilitate identity theft. While this is a frightening prospect, this risk exists for most data repositories in our digital age; it is not unique to healthcare, although healthcare is not immune from it.

Besides hacking to obtain personal and financial information, medical information can be sold. Patients should be informed that their health data will likely be sold. For instance, data may be stored at a company that sells information to advertisers seeking to reach patients with a specific condition or age. An example of this might be a clinical trial recruiting participants with type 2 diabetes in a specific geographical area; the medical organization running the trial may be able to buy lists of prospective recruits and their social media accounts for targeted advertising. Similar tactics are used outside healthcare; for instance, a preschool may buy data from local people with preschool-age children at home. This type of targeted advertising may seem intrusive, but it is widely used and not just in healthcare. It is unlikely that such targeted advertising will be challenged by American legal systems.

Patients may feel comfortable storing their data with physicians, hospitals, and healthcare organizations. Still, they may feel far less comfortable if social media companies or businesses get access to that same data [45]. Physicians should explain who might have access to their data and for what purpose and that wearable owners have little to no say in who has access to their anonymized data.

Some patients may resist sharing data, which is increasingly untenable in our modern internet era but deserves respect. It may be important for such patients to provide the information and then allow them to decide whether using a wearable device is worth the risk. In other words, some patients may feel that using a wearable device or telehealth app is too risky. Thus, in discussing the issue with patients, clinicians must strike the right balance between informing the patient about real risks without unduly alarming them and possibly depriving them of the benefits of wearable systems (Table 3).

**Table 3:** Key considerations in discussing the risk of wearables and other devices with patients AI: artificial intelligence

Points	Pro	Con
Wearables collect data, and the manufacturer owns this data	Data are often anonymized and de-identified	Data can be breached. Identity theft is possible with some systems
Data may be shared or sold to other organizations, universities, and research centers	Data are often anonymized and de-identified, and such data-sharing may have scientific purposes	Even when data are sold, patients get no remuneration
Data may be stored in any number of locations, including overseas. The privacy laws of the place where the data are located are the ones that are in force	Data are often anonymized and de-identified	Patients most likely will not be able to find out where their data are stored. Patients will not be able to remove their data or prevent their data from being stored in specific locations or databases
Health data are being used for AI and other systems to improve healthcare	Your data may be valuable to help build better systems	Patients will not be recognized or compensated for the use of their data
Wearables and their manufacturers may not have as robust security as other organizations, for example, credit card companies or banks	Systems to protect against identity theft, such as online services, may provide a degree of protection	No form of identity protection is fool-proof, and vigilance is recommended

Can clinicians make things better?

Numerous proposals have been discussed to better protect patient privacy. For example, it has been proposed that noise or extraneous information could be artificially added to certain patient data, making it more difficult to re-identify [48]. The drawback to this approach is that it would work on only one aspect of the data, for instance, respiration rate or serum glucose, and not on the totality of information. It is possible that this noise-type security measure could be further refined and improved. Another proposal asks that those collecting wearable data not report individual data but rather report ranges; however, such an approach might limit the utility of these data for certain types of research [27]. Access to de-identified data could be restricted, but this may end up restricting access to valuable data to deserving entities.

Cybersecurity is a global concern and impacts most industries, although healthcare has lagged behind other industries in implementing robust security. Financial institutions, credit card companies, and cell phone services offer tighter security than most medical databases. This is paradoxical since medical data enjoy exalted privacy rights outside the cyber realm. Still, data protections for medical data are limited, making wearables and other such data a particularly inviting target [49]. New initiatives, regulations, and even legislation are needed to bolster patient security and better countermeasures to meet attacks [50].

The discussion about data privacy and security likely will begin with the healthcare professional, who should alert patients to potential concerns without unduly alarming them. Just as informed consent alerts patients to the risks and benefits of medications or procedures, clinicians should inform patients that even the most popular and seemingly harmless wearables are vulnerable to hacking and that their data may be collected, stored, shared, and even sold without their knowledge or consent. Many patients are unaware that their data has value or that seemingly benign medical information may be hacked in identity fraud schemes. Broader public awareness of these risks may expedite legislation and reforms to harden medical data, particularly as so much data is now being driven directly by patients into the healthcare system. The burgeoning use of wearables speaks to a desire of patients to be more health conscious, more proactive in their own lifestyle choices, and more empowered in healthcare. By and large, wearable device users will exhibit a degree of healthcare literacy that exceeds that of the average patient. The lacking element may be digital literacy, the next frontier in patient education.

Healthcare professionals should be at the forefront of explaining the risks and benefits of wearables to patients, many of whom are unaware that the manufacturer owns wearable data or why such companies are eager to collect vast amounts of medical data. Patients may erroneously think that their data are stored only in their devices and have no intrinsic worth to third parties. Finally, people using wearables may not be aware that data hacks can compromise their personal data and expose them to identity theft.

None of these facts necessarily preclude the use of wearables, and there are mitigations against these potential risks. What is lacking is the willingness of healthcare professionals to bring up the unexpected topic of data security with their patients, to explain things frankly, and to field questions about medical privacy. Certain aspects of wearable data privacy may not be subject to change or mitigation: the data belong to the manufacturer, the data can be sold or shared at will, the de-identification of data is not foolproof, and laws governing how medical data are to be handled vary widely among states and nations. These issues cannot be solved at the healthcare system level; they are political topics. However, clinicians should still be able to discuss them with patients.

Some patients may dismiss these concerns, which is their right. However, it is important that clinicians inform them of these risks. Health literacy today must encompass a degree of digital literacy as well. This is not as daunting as it may seem. People of all ages routinely use digital applications for their financial transactions, business emails, and social communications; these systems are not as foreign or fearful as they seem. Younger patients, in particular, have grown up with internet-based tools. The biggest drawback is that wearables are so ubiquitous and user-friendly that many patients may not consider that they are vulnerable to hacking and that the data they record on these systems belongs to the manufacturers. Thus, healthcare professionals must be prepared to explain these risks to patients.

## Conclusions

Wearable devices that collect PGHD have the potential to disrupt medicine and bring about many beneficial advancements, such as virtual physician’s assistants, digitized clinical trials, digital twins for drug development, and a better understanding of health and medical trends in the form of large datasets of continuous real-world data. Wearable devices are popular and ubiquitous, but they may pose risks to patients in the form of data privacy, confidentiality, and security. While medical data are highly protected, the systems, laws, and regulations to secure them are not robust or well-developed. Even the question of medical data ownership is not entirely clear. Clinicians must elevate digital literacy and healthcare literacy among patients to ensure that patients know the risks and benefits of using wearable devices.
